# Digital Image Processing and Convolutional Neural Network Applied to Detect Mitral Stenosis in Echocardiograms: Clinical Decision Support

**DOI:** 10.3390/jimaging11080272

**Published:** 2025-08-14

**Authors:** Genilton de França Barros Filho, José Fernando de Morais Firmino, Israel Solha, Ewerton Freitas de Medeiros, Alex dos Santos Felix, José Carlos de Lima Júnior, Marcelo Dantas Tavares de Melo, Marcelo Cavalcanti Rodrigues

**Affiliations:** 1 Postgraduate Program in Mechanical Engineering, Federal University of Paraíba, João Pessoa 58051-900, PB, Brazil; jfmf@academico.ufpb.br (J.F.d.M.F.); israel.solha@academico.ufpb.br (I.S.); 2 Center of Medical Science, Federal University of Paraíba, João Pessoa 58051-900, PB, Brazil; ewerton.freitas@academico.ufpb.br (E.F.d.M.); marcelot@alumni.usp.br (M.D.T.d.M.); 3 National Institute of Cardiology, Rio de Janeiro 22240-006, RJ, Brazil; alex.felix.ext@dasa.com.br; 4 Department of Mechanical Engineering, Federal University of Paraíba, João Pessoa 58051-900, PB, Brazil; jclj@academico.ufpb.br (J.C.d.L.J.); mcr@academico.ufpb.br (M.C.R.)

**Keywords:** mitral stenosis, digital image processing, convolutional neural network, transesophageal echocardiography, augmentation

## Abstract

The mitral valve is the most susceptible to pathological alterations, such as mitral stenosis, characterized by failure of the valve to open completely. In this context, the objective of this study was to apply digital image processing (DIP) and develop a convolutional neural network (CNN) to provide decision support for specialists in the diagnosis of mitral stenosis based on transesophageal echocardiography examinations. The following procedures were implemented: acquisition of echocardiogram exams; application of DIP; use of augmentation techniques; and development of a CNN. The DIP classified 26.7% cases without stenosis, 26.7% with mild stenosis, 13.3% with moderate stenosis, and 33.3% with severe stenosis. A CNN was initially developed to classify videos into those four categories. However, the number of acquired exams was insufficient to effectively train the model for this purpose. So, the final model was trained to differentiate between videos with or without stenosis, achieving an accuracy of 92% with a loss of 0.26. The results demonstrate that both DIP and CNN are effective in distinguishing between cases with and without stenosis. Moreover, DIP was capable of classifying varying degrees of stenosis severity—mild, moderate, and severe—highlighting its potential as a valuable tool in clinical decision support.

## 1. Introduction

Cardiovascular diseases (CVDs) are prevalent, associated with a high mortality rate, and continue to increase globally. The number of CVD cases nearly doubled from 271 million (95% uncertainty interval (UI): 257 to 285 million) in 1990 to 523 million (95% UI: 497 to 550 million) in 2019. Concurrently, the number of deaths attributed to CVDs has steadily risen from 12.1 million (95% UI: 11.4 to 12.6 million) in 1990 to 18.6 million (95% UI: 17.1 to 19.7 million) in 2019 [1]. In 2021, the incidence of valvular heart disease was reported at 64 cases per 100,000 person-years, with aortic stenosis (47.2%), mitral regurgitation (24.2%), and aortic regurgitation (18.0%) accounting for the majority of valve diagnosis [2]. The mitral stenosis incidence was 2.5%—higher in females (incidence rate: 2.3 versus 1.5) [3].

It is known that among the four heart valves, the mitral valve (MV) is the most affected by pathological alteration process like mitral stenosis (MS), which can be caused by rheumatic fever following an untreated streptococcal infection, such as strep throat. In developing countries, MS remains relatively prevalent, particularly among young women, and may first present as a clinical concern during pregnancy, a period characterized by a significant increase in blood volume due to the presence of the fetus [4]. An analysis of data from the Global Rheumatic Heart Disease Registry showed that 54.8% of Latin American women have MS [5].

Echocardiography provides a visualization of anatomical features, including leaflet thickening and restricted opening resulting from symmetrical fusion of the valve commissures (edges) [6]. However, formal training guidelines for cardiologists acknowledge the importance of experience in accurately interpreting such findings in echocardiograms. Consequently, foundational training in cardiology may be insufficient for achieving a high level of proficiency in echocardiographic interpretation [7,8].

Valve disease typically attracts the clinician’s attention due to the presence of stenosis, insufficiency (also referred to as regurgitation or incompetence), or a combination of both. Stenosis is characterized by failure of a valve to open fully, thereby obstructing forward blood flow [9]. This pathological process alters the normal architecture of the valve, resulting from changes in the leaflet fibers, the formation of blood vessels in the region, and an increased deposition of collagen and other cellular components within the valve tissue [6].

In this context, numerous studies have emerged, aimed at aiding medical diagnosis through the development of tools for the automatic measurement and classification of heart diseases. Alkhodari and Fraiwan [10]; Joshi et al. [11]; and Bhardwaj, Singh, and Joshi [12] employed convolutional neural networks (CNNs) to classify various heart conditions, including aortic stenosis, mitral regurgitation, mitral stenosis, and mitral valve prolapse, utilizing heart sounds (phonocardiograms) as input data. Specifically, Alkhodari and Fraiwan [10] implemented a combination of CNNs and recurrent neural networks based on bidirectional long short-term memory (Bi-LSTM). Joshi et al. [11] utilized two deep learning architectures: a one-dimensional CNN and a spectrogram-based two-dimensional CNN. Bhardwaj, Singh, and Joshi [12] applied a two-dimensional CNN to the time–frequency representation of phonocardiogram signals for multi-class classification.

Cohen-Shelly et al. [13] used standard 12-lead electrocardiograms as input data for a convolutional neural network (CNN) to classify patients into two groups: those with moderate to severe aortic stenosis and those with mild or no aortic stenosis. The severity of aortic stenosis was categorized as moderate to severe based on the following criteria: peak velocity ≥ 3.0 m/s, mean gradient ≥ 20 mmHg, dimensionless velocity index ≤ 0.35, or aortic valve area ≤ 1.5 cm^2^. The artificial intelligence model was trained on 129,788 examinations, validated on 25,893 examinations, and tested on 102,926 randomly selected examinations.

Ulloa-Cerna et al. [14] trained a convolutional neural network (CNN) using 12-lead electrocardiograms as input data to predict the presence or absence of various heart diseases. Some of the results for mitral stenosis were as follows: prevalence, 0.3 (95% confidence interval: 0.2–0.3); area under the receiver operating characteristic curve, 0.918 (95% confidence interval: 0.905–0.930); and area under the precision–recall curve, 0.039 (95% confidence interval: 0.036–0.044).

Wegner et al. [15] were among the first to validate view classification using convolutional neural networks (CNNs) in patients with congenital or structural heart disease through echocardiographic images. Zhang et al. [16] employed image segmentation techniques to detect three distinct diseases from echocardiograms: hypertrophic cardiomyopathy, cardiac amyloidosis, and pulmonary arterial hypertension. They utilized image segmentation to train a CNN for the identification of cardiac chambers.

The present study aimed to apply digital image processing techniques and develop a convolutional neural network (CNN) capable of classifying videos from three-dimensional transesophageal echocardiograms (3D TEE) into cases with mitral stenosis and those without, thereby serving as a diagnostic tool for this condition. To achieve this objective, echocardiogram videos were acquired from experts in the field. The image segmentation method selected for this study was the single-band fixed threshold technique.

Furthermore, the acquired videos underwent data augmentation techniques to increase the volume of training data for the convolutional neural network (CNN). A total of 30 echocardiogram videos were collected, each originating from a distinct examination and patient. Ultimately, the developed CNN model achieved an accuracy of 92% in detecting mitral stenosis from the echocardiogram video files.

## 2. Materials and Methods

This study was conducted in four stages: (1) the acquisition of echocardiogram examinations in video format; (2) the application of digital image processing (DIP) to classify the videos into cases with and without mitral stenosis, serving as a preprocessing tool for the convolutional neural network (CNN); (3) the utilization of data augmentation techniques to enhance the volume of training data for the CNN; and (4) the development of a CNN to detect cases with and without mitral stenosis from the echocardiogram videos.

### 2.1. Data Acquisition

This study involved thirty 3D transesophageal echocardiogram examinations, each from a different patient. These exams were performed and made available by two experts in the field, with authorization obtained from the Ethics Committee in Research (Technical opinion no. 3.858.742—CEP/CCM/UFPB).

Transesophageal echocardiography (TEE) is a critically important imaging modality in cardiovascular diagnostics. The proximity of the esophagus to the heart and the great vessels provides an excellent ultrasonic window for imaging [17]. Compared to transthoracic echocardiography, the ultrasound in TEE traverses a much shorter path without obstructions, resulting in clearer images and more accurate information, free from distortions.

This study employed a retrospective, cross-sectional, and observational design, with data acquisition conducted in accordance with the recommendations of the American Society of Echocardiography [18].

Echocardiographic images were obtained through planimetry performed by multiplanar reconstruction, encompassing both ventricular and atrial views. This was achieved using EchoPac v.204 for GE devices and directly on the Epiq 7 device for Philips equipment during the echocardiographic examinations.

Using transesophageal echocardiography, images were acquired in a section of the mid-esophagus focused on the mitral valve (MV). Planimetry was performed utilizing either one-beat or multi-beat techniques to achieve improved spatial and temporal resolution.

The images were post-processed using multiplanar reconstruction tools and navigation in rendered mode, specifically in an en face view of the valve orifice, from both the ventricular and atrial perspectives. This approach was employed to achieve better alignment and accurate measurement of the largest diastolic area of the mitral valve orifice.

Manual tracing of the mitral valve orifice edges was performed offline by experienced examiners to determine the maximum diastolic valve area, using the appropriate workstation for each vendor (Philips and GE equipment). No significant gain adjustments were made during post-processing, as such adjustments could either underestimate (high gain) or overestimate (low gain or presence of dropout) the valve area. Instead, this study accepted the adjustments made prior to the three-dimensional acquisition, which generally corrected for any distortions due to excessive or insufficient gain.

Finally, the examinations were saved in video format with an .avi extension. Consequently, a total of 30 videos were recorded, each corresponding to a different patient. Each video was of sufficient duration to capture the opening and closing of the mitral valve.

### 2.2. Digital Image Processing

In general, patients with a mitral valve area (MVA) between 4 and 5 cm^2^ are considered asymptomatic. Those with an MVA ranging from 1.5 cm^2^ to 4 cm^2^ are classified as having mild stenosis, while patients with an MVA between 1 cm^2^ and 1.5 cm^2^ exhibit moderate stenosis. Severe stenosis is defined as an MVA of less than 1 cm^2^ [19]. In this case, the objective of the DIP was to detect and calculate the area of maximum mitral valve opening for each video, facilitating the classification of the 30 saved echocardiogram videos. The videos were categorized into four groups: severe mitral stenosis, moderate mitral stenosis, mild mitral stenosis, and cases without mitral stenosis.

**Figure 1:**
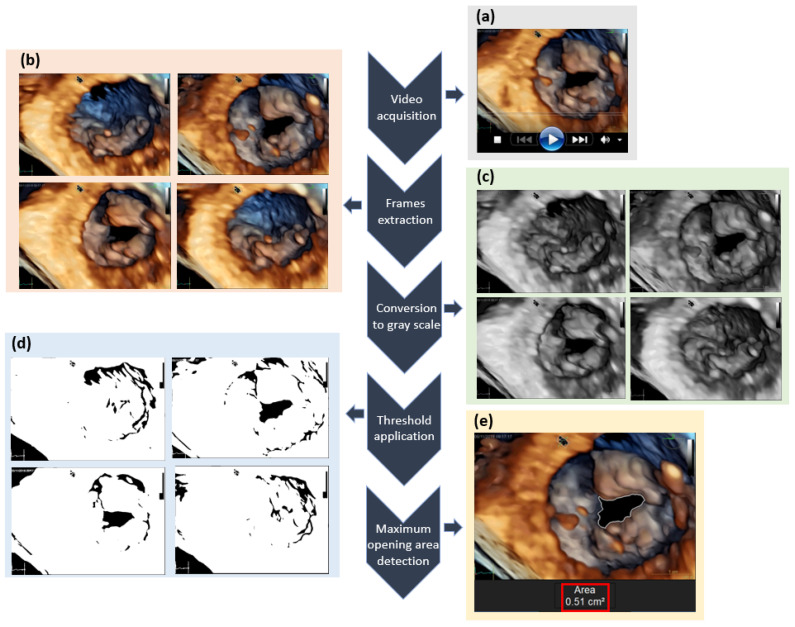
Flowchart of the digital image processing applied: (**a**) video acquisition; (**b**) frame extraction; (**c**) conversion to grayscale; (**d**) threshold application; (**e**) maximum opening area detection.

The entire procedure implemented in the digital image processing (DIP) is illustrated in the flowchart presented in Figure 1. Initially, the echocardiogram videos were saved in .avi format (Figure 1a). Subsequently, frames were extracted from each video, transforming the initial video into a collection of images (Figure 1b). Each frame was converted from RGB (red, green, and blue) to grayscale (Figure 1c). Following this, each grayscale frame was converted to a binary scale using the single-band fixed threshold method (Figure 1d). With the frame in binary scale, it became possible to identify the contours of the images and highlight the frame that exhibited the largest valve opening area (Figure 1e).

Video refers to pictorial (visual) information that encompasses both still images and time-varying images. A still image represents a spatial distribution of intensity that remains constant over time. In contrast, a time-varying image is characterized by changes in the spatial intensity pattern over time [20].

An image can be defined as a two-dimensional function, *f*(*x*,*y*), where *x* and *y* represent spatial coordinates in a plane, and the amplitude *f* at any given pair of coordinates (*x*,*y*) is referred to as the intensity. When *x*, *y*, and the amplitude values of *f* are all finite, discrete quantities, the image is classified as a digital image [21].

In this context, the frames of each video were initially extracted (Figure 1b), and each frame was subsequently converted from the RGB (red, green, and blue) color scale to grayscale (Figure 1c). For this conversion, the luminance method was employed, which does not account for the greater sensitivity of the human eye to the green color. As a result, the G channel is given a larger weight compared to the R and B channels [22]:

Luminance = 0.2989 × R + 0.5870 × G + 0.1140 × B(1)

Among the numerous tools available for digital image processing, the single-band fixed threshold method was selected to convert the frames from grayscale to binary scale and to identify the area of maximum valve opening.

The single-band fixed threshold method is a relatively straightforward approach to segmenting an image into regions of similarity. The fundamental principle involves grouping pixels within a specified range of gray levels into a predetermined set. The simplest implementation of this technique involves first normalizing the image so that the pixel values range between 0 and 1, where *v* represents the pixel value at coordinates (*i*,*j*). Single-band fixed thresholding transforms an image of this type into a binary form, consisting solely of 0s and 1s, through the application of the following processes [23]:

(2) $$0\leq v_{ij}\leq 1$$

If

(3) $$v_{ij}^{in}>threshold$$

(4) $$v_{ij}^{out}=1$$

Else

(5) $$v_{ij}^{out}=0$$

In this method, 0 < threshold < 1. In certain scenarios, it may be advantageous to retain the gray level variations that occur above the threshold. In the grayscale representation, 0 corresponds to black, while 255 corresponds to white [23]. Consequently, in the grayscale context, the threshold value must be greater than zero and less than 255.

The single-band fixed threshold technique yields a binary image in which pixels with a value of one indicate that they are part of the object under investigation, while pixels with a value of zero represent the background or are not included in the object [24].

The threshold applied to a grayscale image is crucial for generating contours within the image. For instance, if the threshold value is set at 20, all gray pixels with values lower than 20 will be converted to black, while the remaining pixels will be transformed into white (Figure 1d). The transition between white and black pixels facilitates the generation of contours, which are subsequently rendered in RGB scale to highlight them within the original image (Figure 1e).

In the present study, which utilizes video footage depicting the opening and closing of the mitral valve, it is essential to differentiate between the surfaces representing the heart and mitral valve and the surrounding areas that do not. In echocardiogram videos, the surfaces are rendered in various colors, while empty spaces are depicted in black. Consequently, when the mitral valve is closed, the image’s solid body will appear filled with different colors. In contrast, when the mitral valve is open, a black region will emerge between the colored surfaces.

Thus, the transition region between colored areas and black is identified through the application of the threshold method, which delineates a contour as a result. With this contour established, it becomes possible to identify all pixels constituting the valve opening, enabling the determination of the area of maximum valve opening and the calculation of its numerical value (Figure 1e).

The area of the mitral valve opening was calculated by multiplying the total number of pixels constituting the highlighted opening area, as delineated by the contour, by the area value assigned to each pixel. The area value of each pixel varies across different videos. For videos acquired using Philips equipment, the height and width of each pixel are determined based on the distance between the centroids of two consecutive green points, measured in centimeters or millimeters. In contrast, for videos obtained with GE equipment, the width of each pixel is defined by a straight line located in the lower right corner of the video. It is assumed that the pixel is symmetrical, thereby allowing for the height value to be used as well.

**Figure 2:**
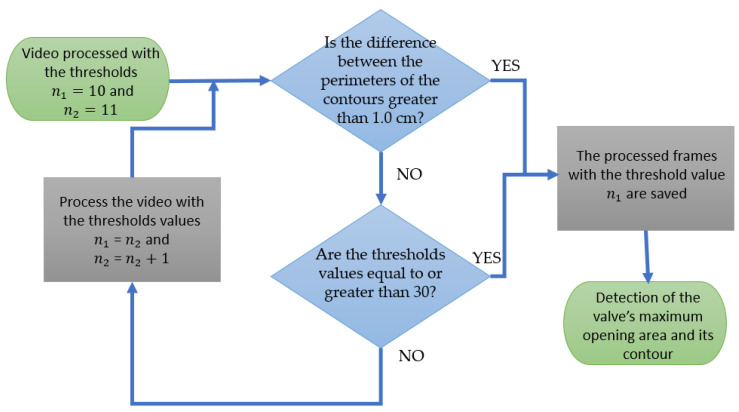
Flowchart of the method used to detect the maximum valve opening area.

For the application of the single-band fixed threshold technique, it was necessary to carry out the manipulation described in the flowchart in Figure 2, considering that there are 255 possible threshold values to be applied to frames in gray scales.

The video is initially processed with a threshold value (*n*) set to 10, allowing selection of the frame that captures the largest valve area. This process is repeated by incrementing the threshold to *n* + 1, each time selecting the frame with the largest observed valve area. This incremental approach continues until a perimeter difference greater than 1.0 cm is detected between the contours of maximum valve areas obtained with thresholds *n* + 1 and *n*. Among the resulting frames, the one capturing the maximum valve opening and displayed as the final output corresponds to the penultimate threshold analyzed, as the last threshold exceeded the 1.0 cm perimeter difference criterion.

Thus, for each exam, threshold values ranging from 10 to 30 are applied sequentially. The analysis is halted either when the perimeter difference between contours of maximum valve openings exceeds 1.0 cm or when the threshold reaches the upper limit of 30. This approach ensures that the selected frame corresponds to the optimal threshold, accurately highlighting the largest valve opening without overextending the contour perimeter difference criteria.

Setting the perimeter difference threshold to 1.0 cm was intended to mitigate abrupt variations in the contour delineations between successive thresholds. This value serves to prevent disproportionate expansions or reductions in the contour, ensuring a consistent and gradual transition. By maintaining this constraint, the process enhances contour accuracy and stability, yielding a reliable measure of the maximum valve opening.

**Figure 3:**
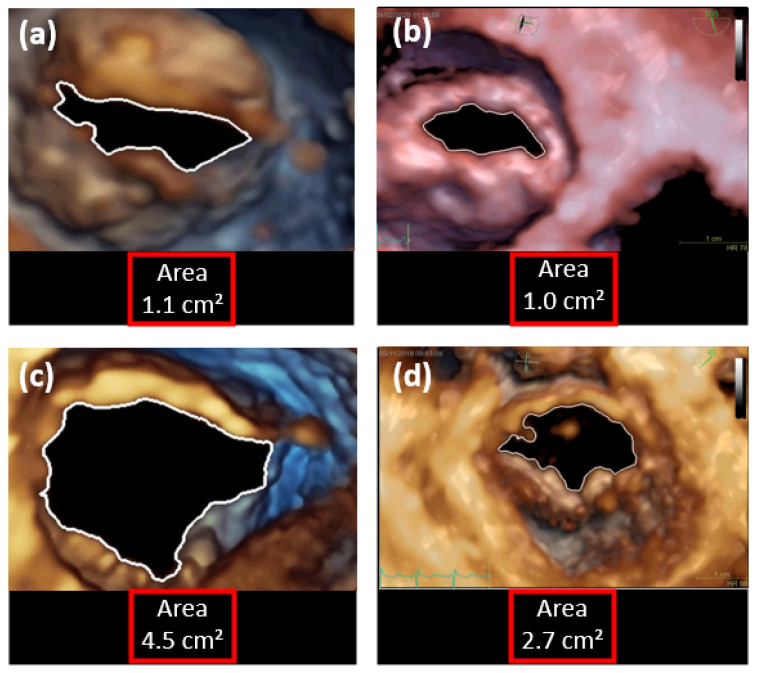
Examples of DIP results. (**a**,**b**) Cases with moderate mitral stenosis; (**c**) case without stenosis; (**d**) case with mild mitral stenosis.

Figure 3 demonstrates the outcomes of the digital image processing technique across four distinct echocardiogram exams. Each image displays the frame capturing the maximum mitral valve opening for a different patient, with the calculated area shown. The contours of these openings were emphasized with a solid white line to enhance visibility. The efforts of Barros Filho et al. [25] marked the initial steps toward creating a software solution that automates this digital image processing step, featuring an accessible and user-friendly graphical interface for clinicians and researchers alike.

The videos displayed in Figure 3 illustrate different classifications of mitral stenosis based on their maximum mitral valve opening areas. Specifically, the videos in Figure 3a,b are categorized as cases of moderate mitral stenosis, with maximum opening areas ranging from 1.0 cm^2^ to 1.5 cm^2^. In contrast, Figure 3c represents a case without stenosis, as it exhibits an opening area between 4.0 cm^2^ and 5.0 cm^2^. Finally, Figure 3d is classified as a case of mild mitral stenosis, with an opening area between 1.5 cm^2^ and 4.0 cm^2^.

Finally, the DIP results for the 30 exams acquired are illustrated in Table 1. It can be seen, therefore, that out of the 30 exams, 8 (26.7%) were classified as cases without mitral stenosis, 8 (26.7%) as cases with mild mitral stenosis, 4 (13.3%) as cases with moderate mitral stenosis, and 10 (33.3%) as cases with severe mitral stenosis. All 30 videos with their respective classifications from DIP were used in the development of the CNN and the image data augmentation.

### 2.3. Augmentation

Data augmentation is a technique used to artificially increase the size of the training dataset. This is important because, for many complex real-life problems (e.g., medical datasets), only a limited training dataset may be available. Several data augmentation operations are available, such as cropping, rotations, flipping, translations, contrast adjustment, scaling, and more [26].

The methodology for image data augmentation in this study employed a comprehensive approach to enhance the diversity of the training dataset. This began with defining a set of parameters to guide the transformations applied to the images. Specifically, the rotation parameter enabled random rotations within a range of 40 degrees. Additionally, translation parameters allowed for horizontal and vertical shifts of up to 20% of the image’s width and height, respectively.

The methodology included parameters for introducing random shear and zoom effects, which enhanced the spatial variability of the images. Horizontal flipping was incorporated to improve the model’s ability to generalize across different orientations. The nearest neighbor interpolation method was employed to fill in any newly created pixel values after transformations, ensuring smooth transitions and preserving the integrity of the images.

Images were extracted from the video files using a custom class that employed video capture functionality to retrieve frames. Each frame was processed individually to prepare it for augmentation. The images were converted from BGR color space to RGB format to ensure compatibility with the augmentation process. Subsequently, the images were adjusted to include a batch dimension, as required by the augmentation framework.

**Figure 4:**
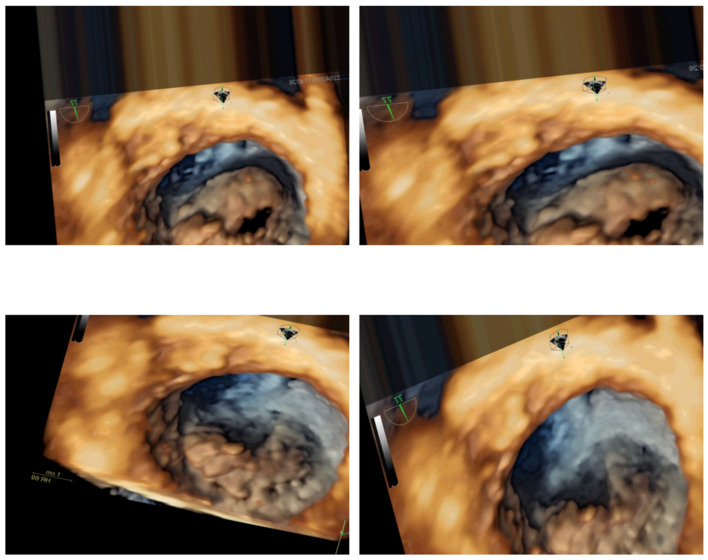
Examples of generated images for two distinct frames.

The augmentation was performed iteratively for each frame in the video collection. For each original image, a specified number of augmented images were generated and saved to a designated directory. The number of augmented images to generate per frame was determined by a parameter defined in the augmentation function. Each augmented image was saved in a structured format that included a prefix indicating the class and a unique count identifier, facilitating subsequent data management. In this study, to achieve a better hardware usage/result ratio, six additional images were generated for each individual frame. Some examples can be seen in Figure 4.

After generating the augmented images, the dataset was split into training and testing subsets. This was accomplished by randomly selecting a specified proportion of images to form the test set, while the remaining images constituted the training set (30% for testing and 70% for training). The split was designed to maintain a balance between the two datasets, ensuring that the model would be trained on a diverse range of augmented images while retaining a separate unseen set composed of two videos from each class (with and without stenosis) for validation purposes.

For the trained model, all frames from the video collection of exams from patients with stenosis were divided into training and testing subsets, as were the frames from the exams of patients without stenosis. In this way, the CNN was trained not only with frames showing the presence of the condition but also with all frames from each, aiming to identify patterns throughout the entire video and maximize accuracy.

The implementation also included the establishment of necessary directory structures for training and testing images, thus facilitating efficient data access during the model training phase. Each category of images was organized into its respective folder, allowing for seamless integration with data loading utilities that can automatically retrieve images based on their directory structure.

### 2.4. Convolutional Neural Network

A convolutional neural network (CNN) is a type of artificial neural network (ANN) characterized by a deep feedforward architecture and exceptional generalization capabilities compared to other networks with fully connected layers [24]. The CNN architecture implicitly assumes that the input is image-like, allowing specific properties to be encoded within the design. In particular, convolutions facilitate the capture of translation invariance [27].

This, in turn, makes the forward function more efficient, significantly reduces the number of parameters, and thus makes the network easier to optimize and less dependent on dataset size [27].

In contrast to regular neural networks, CNN layers have neurons organized across several dimensions: channels; width; height; and, in the simplest 2D case, the number of filters. Like a multilayer perceptron, a convolutional neural network consists of a sequence of layers, where each layer transforms the activations or outputs of the previous layer through another differentiable function [27].

Additionally, the CNN contains a set of convolutional kernels (also known as filters), which convolve with the input image (N-dimensional matrix) to produce an output feature map [26].

The CNN model structure follows a typical architecture used for image classification tasks. It consists of several layers: initially, there are four convolutional layers, each followed by a max-pooling layer, which reduces the spatial dimensions of the feature maps and helps with translation invariance. Each convolutional layer uses a 3 × 3 kernel with ReLU activation, gradually increasing the number of filters from 32 to 256, allowing the model to learn more complex patterns at deeper levels. After these convolutional and pooling layers, the model flattens the output to a 1D vector and passes it through a fully connected dense layer with 512 units and a ReLU activation function. Finally, the output layer consists of two units (for binary classification) with a softmax activation function, which outputs the class probabilities, chosen for its advantages in classification output [28].

The ReLU activation function was chosen because it is particularly well suited for larger models, as it accelerates the training process and enhances the learning of features that are more effective for pattern recognition [29]. The model was compiled using the Adam optimizer and a classification-appropriate loss function. Training was conducted over a specified number of epochs with a defined batch size, leveraging the prepared image data for both training and validation datasets.

The Adam optimizer was selected for training due to its various advantages, including adaptive learning rates for each parameter, which help stabilize the training process. This optimizer combines the benefits of two other extensions of stochastic gradient descent: AdaGrad, which adapts the learning rate to the parameters, and RMSProp, which adjusts the learning rate based on the average of recent gradient magnitudes. Consequently, Adam often results in faster convergence and improved performance, particularly when training deep networks like CNNs. These characteristics are especially beneficial in scenarios with sparse gradients or noisy objectives [30].

The model was trained over ten epochs with a batch size of 100 images, using the prepared image data for both training and validation datasets. Its performance was evaluated based on accuracy in classifying validation dataset images during training. The training process involved monitoring both training and validation accuracy to ensure the model did not overfit. The final trained model was saved for future inference tasks and broader application.

All frames from each video, regardless of whether they showed the presence of the condition, were used in both the training and testing processes. By adopting this approach, the CNN was trained on the entire set of frames from each video, enabling it to identify patterns not only when the condition was present but also throughout the entire video content, with the goal of maximizing the model’s accuracy.

**Figure 5:**
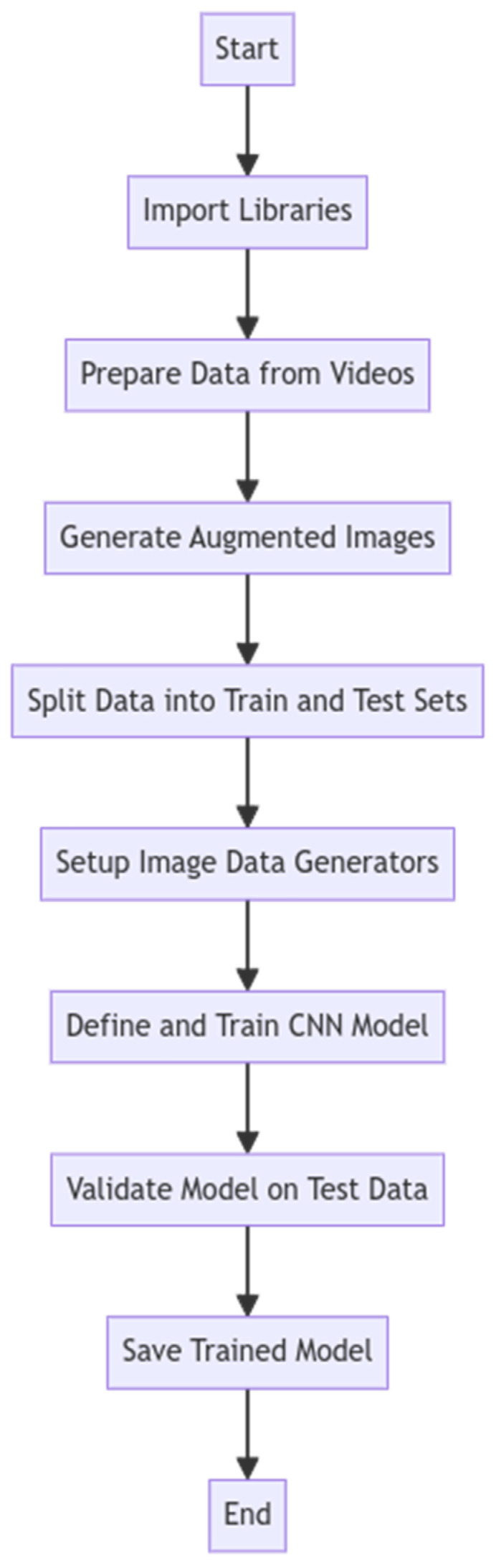
Flowchart of the algorithm structure.

The trained model’s classification capabilities were further evaluated through a dedicated function enabling frame-by-frame predictions on new video files. This function accepted the video path and model path as inputs, loading the trained CNN model to process each frame sequentially. Each frame was resized to the model’s input dimensions and normalized before being passed through the model for prediction. Predictions could be returned in raw format for further analysis or processed to generate a binary output indicating the presence or absence of mitral stenosis. This systematic approach provided a robust framework for both training the CNN model and evaluating its performance in classifying video frames. The general structure of the developed algorithm is illustrated in Figure 5.

The CNN was initially developed to classify videos into four categories: severe mitral stenosis, moderate mitral stenosis, mild mitral stenosis, and cases without mitral stenosis. However, the model demonstrated low accuracy in distinguishing between the different degrees of stenosis severity. Despite applying data augmentation techniques, the number of acquired exams remained insufficient to effectively train the model for this purpose. As a result, the CNN was ultimately trained to classify the videos into two groups: cases with stenosis (including severe, moderate, and mild mitral stenosis) and cases without stenosis.

## 3. Results

The training of the convolutional neural network (CNN) model for classifying echocardiogram videos as either with or without mitral stenosis yielded promising results over eleven epochs. Initially, the model achieved around 68% accuracy in the first epoch, which rose significantly to 96% by the seventh epoch. Only the first seven epochs were relevant, as the early stopping callback, implemented to prevent overfitting by monitoring test loss progression, halted training early and saved the best weights.

**Figure 6:**
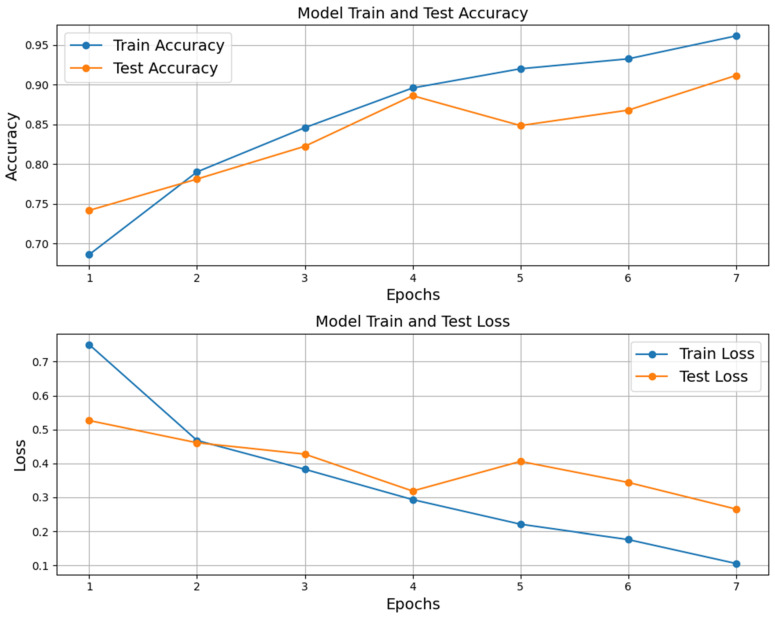
Training and testing accuracy and loss for the trained model.

The loss function also showed a steady decline, starting at 0.72 and decreasing to 0.11, indicating effective learning. Test accuracy followed a similar trend, rising from 74% in the first epoch to 92% by the seventh epoch. This simultaneous improvement in both training and test metrics suggests that the model not only learned effectively from the training dataset but also generalized well to unseen data, as illustrated in Figure 6.

**Figure 7:**
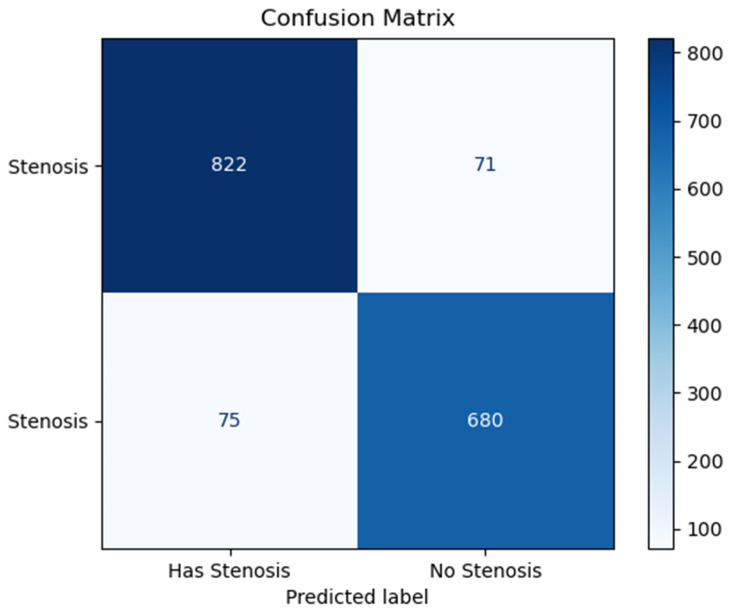
Confusion matrix for the model in a test with 1648 test samples.

The confusion matrix generated from the test dataset provides further insight into the model’s performance. Out of 1648 total test samples (augmented images from original frames), 822 instances were accurately identified as echocardiograms of patients with stenosis, and 680 were correctly classified as echocardiograms without stenosis. The model successfully identified 822 images with only 75 false positives, as illustrated in Figure 7. The high true positive rate (TPR) and low false negative rate (FNR) demonstrate the model’s effectiveness in detecting cases of mitral stenosis, which is essential for its clinical applicability in cardiology.

To further validate the model’s classification abilities, it was applied to five videos that were not included in the training and testing processes (thus unknown to the model). All predictions aligned with the expected outcomes: three videos were classified as “No stenosis” and two as “Has stenosis”. Each of these predictions corresponded accurately to the true conditions of the videos, demonstrating the model’s robustness in real-world applications. The accuracy of predictions in these unknown cases reinforces the efficacy of the CNN in classifying complex patterns in echocardiograms, thereby supporting its potential utility in clinical settings for the automated detection of mitral stenosis.

The trained model was used to evaluate the entire test dataset, achieving an accuracy of 92% with a loss of 0.26. When applied sequentially to the videos used for testing, training, and validation, the trained CNN successfully classified 92% of the echocardiograms concerning the presence or absence of mitral stenosis.

In summary, the training, validation, and testing of the CNN model demonstrate strong performance in distinguishing between echocardiograms with and without mitral stenosis. The model’s high accuracy rates during training and validation, coupled with favorable outcomes from the confusion matrix and real-world testing, indicate that it can serve as a reliable tool for assisting clinicians in diagnosing mitral stenosis from video data. Further research and optimization could enhance its capabilities and expand its applications in cardiology diagnostics.

**Figure 8:**
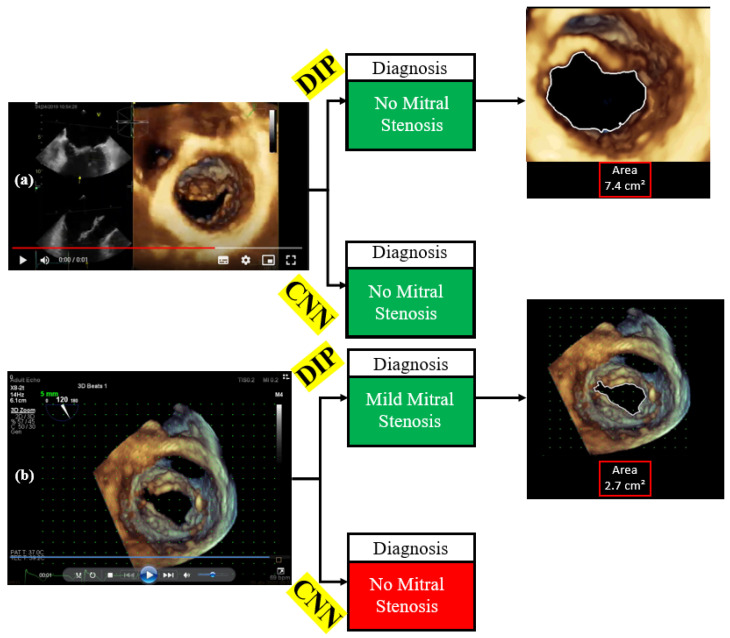
Comparison between DIP and CNN results. (**a**) DIP and CNN detected the case without mitral stenosis. (**b**) CNN did not correctly classify the clinical case of mild mitral stenosis.

Finally, the CNN results were compared with the DIP results. Two examples of these comparisons can be seen in Figure 8.

In Figure 8a,b, the echocardiogram videos were subjected to both digital image processing (DIP) and convolutional neural network (CNN) analyses. In Figure 8a, both DIP and the CNN diagnosed the absence of mitral stenosis. The DIP analysis additionally provided results that highlighted the frame with the largest valve opening area, including the contour and the value of the patient’s maximum valve opening area. In contrast, the CNN only offered the diagnosis as a result.

In Figure 8b, the diagnoses from DIP and the CNN diverged. DIP indicated the presence of mild mitral stenosis, while the CNN diagnosed the absence of mitral stenosis. However, the area of maximum valve opening measured at 2.7 cm^2^ clearly indicates that the case in Figure 8b is one of mild mitral stenosis. This suggests that the CNN was unable to detect mild mitral stenosis for this specific exam.

Therefore, when comparing DIP and the CNN, it is evident that the developed CNN was only capable of classifying videos as either case with or without stenosis; as can be seen in Table 2, the number of acquired exams remained insufficient, despite the application of data augmentation techniques, for the CNN to identify a pattern between cases without stenosis and cases with mild stenosis.

In contrast, DIP not only classified cases but also calculated the area of maximum valve opening, highlighted the contour of this maximum opening, and identified the corresponding frame. This additional information provided by DIP aids specialists in making more informed diagnoses.

## 4. Discussion

The works of Alkhodari and Fraiwan [10]; Joshi et al. [11]; and Bhardwaj, Singh, and Joshi [12] utilized phonocardiograms as input data for their CNNs. In contrast, the studies by Cohen-Shelly et al. [13] and Ulloa-Cerna et al. [14] employed electrocardiograms as input data for their CNNs. Meanwhile, our study focused on using echocardiograms as input data for the development of our CNN.

Siqueira et al. [31] conducted a systematic review of studies that applied artificial intelligence to support medical decisions based on the automatic analysis of echocardiogram images. Of the 118 studies evaluated, only 11 obtained echocardiograms from transesophageal examinations. Among these, one study applied the images to a cardiac window view plane, one focused on the quantification and analysis of cardiac functions, and nine were aimed at the detection and classification of heart diseases. Notably, none of these nine studies used CNNs.

Therefore, it can be concluded that our work contributes to the growing body of research on medical decision support that utilizes convolutional neural networks (CNNs) applied to transesophageal echocardiogram exams.

Our study chose to take as an example the work of Zhang et al. [16], who applied image segmentation to train the CNN to identify cardiac chambers. In our case, the image segmentation technique known as single-band fixed threshold was used as a preprocessing tool to classify videos between four cases (no stenosis, mild stenosis, moderate stenosis, and severe stenosis) based on the value of the area of maximum valve opening identified for each exam.

While studies such as Cohen-Shelly et al.’s [13] classified the degree of severity of stenosis considering several parameters, such as peak velocity, mean gradient, dimensionless velocity index, and valve area, ours used only the area of maximum valve opening, considering areas ≥ 4.0 cm^2^ as without stenosis, 1.5 cm^2^ < areas < 4.0 cm^2^ as mild stenosis, 1.0 cm^2^ ≤ areas ≤ 1.5 cm^2^ as moderate stenosis, and areas < 1.0 cm^2^ as severe stenosis.

A limitation of our study was the total number of echocardiogram videos, 30. To address this issue, we employed data augmentation techniques to increase the diversity of the dataset, which was essential for training the CNN to effectively identify patterns associated with the presence or absence of mitral stenosis. Acquiring more exams is essential to improve the CNN’s performance.

Furthermore, this study highlighted that the single-band fixed threshold, considered a very simple method of image segmentation, was effective in classifying videos into cases with or without stenosis. This contrasts with studies such as Shahid and Schizas [32], which employed a technique called Otsu automatic thresholding to track the movement of the mitral valve leaflets, and that of Faraji et al. [33], which used a combination of the circular Hough transform and k-means algorithms to detect the mitral valve orifice and the mid-diastole frame from 2D echocardiogram exams.

In addition, DIP calculated the area of maximum valve opening, highlighted the contour of this maximum opening, highlighted the corresponding frame, and classified the cases into four categories: severe mitral stenosis, moderate mitral stenosis, mild mitral stenosis, and cases without mitral stenosis. In contrast, the developed convolutional neural network (CNN) was only capable of classifying cases into two groups—stenosis and no stenosis.

Both DIP and the CNN contributed to the detection of mitral stenosis in AVI video files, thereby eliminating the reliance on DICOM files, which are often limited in compatibility with available software.

This study aims to demonstrate the valuable contribution of both digital image processing (DIP) and convolutional neural networks (CNNs) in supporting the diagnosis of mitral stenosis. In clinical practice, particularly with patients who have arrhythmias, selecting a video frame at an inappropriate time can significantly impact diagnostic accuracy. By leveraging DIP to determine the severity of the stenosis and CNN with 92% accuracy to classify the presence of the condition, both techniques can work to minimize human errors. These methods provide reliable, consistent decision support, offering specialists an additional tool for comparison with traditional medical measurements and improving the overall diagnostic process.

## 5. Conclusions

The main objective of this study was to apply digital image processing (DIP) and to develop a convolutional neural network (CNN) to serve as decision support for specialists in diagnosing mitral stenosis based on transesophageal echocardiography exams. The CNN model successfully identified 822 images, with only 75 false positives, and was able to correctly classify 92% of the echocardiograms regarding the presence or absence of mitral stenosis.

Both DIP, using the single-band fixed threshold technique, and the CNN were able to classify cases with and without. Therefore, DIP was able to classify different degrees of stenosis severity: mild, moderate, and severe. They can be utilized as decision support tools in the fight against heart disease.

However, DIP presented advantages such as calculating the area of maximum mitral valve opening for each exam.

## Figures and Tables

**Table 1:** Classification of echocardiogram videos based on digital image processing.

Echocardiogram Exam Videos	Area of Maximum Mitral Valve Opening Calculated by DIP	Classification
1	4.2 cm^2^	No Stenosis
2	5.3 cm^2^	
3	5.1 cm^2^	
4	4.0 cm^2^	
5	5.5 cm^2^	
6	4.9 cm^2^	
7	7.4 cm^2^	
8	4.5 cm^2^	
9	2.7 cm^2^	Mild Stenosis
10	1.6 cm^2^	
11	1.6 cm^2^	
12	1.8 cm^2^	
13	3.7 cm^2^	
14	2.6 cm^2^	
15	3.2 cm^2^	
16	1.6 cm^2^	
17	1.0 cm^2^	Moderate Stenosis
18	1.1 cm^2^	
19	1.0 cm^2^	
20	1.1 cm^2^	
21	0.7 cm^2^	Severe Stenosis
22	0.9 cm^2^	
23	0.5 cm^2^	
24	0.4 cm^2^	
25	0.6 cm^2^	
26	0.4 cm^2^	
27	0.7 cm^2^	
28	0.6 cm^2^	
29	0.7 cm^2^	
30	0.7 cm^2^	

**Table 2:** Difference between the DIP and CNN diagnoses.

	DIP	CNN
**Diagnosis**	No Stenosis	No Stenosis
	Mild Stenosis	Has Stenosis
	Moderate Stenosis	
	Severe Stenosis	

## Data Availability

Dataset available on request from the authors.
